# Epidemiological characteristics, virulence potential, antimicrobial resistance profiles, and phylogenetic analysis of *Aeromonas caviae* isolated from extra-intestinal infections

**DOI:** 10.3389/fcimb.2023.1084352

**Published:** 2023-02-24

**Authors:** Yang Song, Li-feng Wang, Kuai Zhou, Shuang Liu, Ling Guo, Li-yan Ye, Jiang Gu, Yan Cheng, Ding-xia Shen

**Affiliations:** ^1^ Department of Clinical Laboratory, Medical School of Chinese PLA, Beijing, China; ^2^ Department of Clinical Laboratory, First Medical Center of Chinese PLA General Hospital, Beijing, China; ^3^ Department of Clinical Laboratory, Xuanhan People’s Hospital, Xuanhan, Sichuan, China; ^4^ Department of Clinical Laboratory, Hainan Modern Women and Children’s Hospital, Haikou, China; ^5^ Department of Clinical Microbiology and Immunology, College of Pharmacy and Medical Laboratory, Army Medical University, Chongqing, China; ^6^ Department of Basic Medical Laboratory, The 980th Hospital of the PLA Joint Logistical Support Force, Bethune International Peace Hospital, Shijiazhuang, China

**Keywords:** *Aeromonas caviae*, phylogenetic analysis, antimicrobial resistance, extra-intestinal infections, virulence

## Abstract

**Objective:**

*Aeromonas caviae* (*A. caviae*) is one of the major etiological agents in human intestinal infections reported to be associated with a broad spectrum of extra-intestinal infections with increasing incidence over recent years. Although previous studies have established its significance as a causative agent of both bloodstream and gastrointestinal infections, the characteristics of A. caviae that cause extra-intestinal infections remain unilluminated.In this single-center retrospective study, we investigated epidemiological characteristics, antimicrobial resistance genes and phenotypes, virulence genes, and phyloevolution of 47 clinical *A. caviae* isolated from patients with extra-intestinal infections from 2017 to 2020.

**Methods:**

*A. caviae* strains were identified by biochemical tests and matrix-assisted laser desorption/ionization-time of flight mass spectrometry (MALDI-TOF/MS), ultimately confirmed to species level by whole-genome sequencing (WGS). Antimicrobial resistance and virulence genes were identified using the Comprehensive Antibiotic Resistance Database (CARD) and the virulence factor database (VFDB), respectively. Phylogenetic analysis of 47 clinical strains was performed by combining with 521 *A. caviae* strains from NCBI database.

**Results:**

*A. caviae* was an opportunistic pathogen in immunocompromised patients, especially those with underlying hepatobiliary diseases and malignancies. 19 out of 47 isolates were identified as multidrug resistance (MDR) strains. Piperacillin-tazobactam, levofloxacin, gentamicin, amikacin with a resistance rate of less than 10% remained as options to treat extra-intestinal infections. 24 out of 47 isolates exhibited non-susceptibility to cephalosporins and cephamycins, all of which carried β-lactamase gene, including *bla*_MOX_, *bla*_PER-3_, *bla*_OXA_, *bla*_NDM_, and *bla*_CphA_. Most stains (98%, 46/47) carried at least one of the virulence genes, but extra-intestinal infections had a low mortality rate. Phylogenetic analysis indicated the risk of nosocomial transmission but revealed no outbreak. However, the emergence of MDR and β-lactamase resistance genes in extra-intestinal isolates of *A. caviae* is becoming an increasing risk to public health and requires attention.

**Conclusions:**

This study strengthen our understanding of *A.caviae* isolated from extra-intestinal infections. It may contribute to the management of extra-intestinal infections as well as the prevention and control of drug resistance.

## Introduction

*Aeromonas* spp. belongs to *Aeromonadaceae*, a kind of Gram-negative, rod-shaped, facultative anaerobic bacteria, which are ubiquitous in the aquatic environment, soil, fish, animals and foodstuffs (Janda & Abbott, 2010; Fernandez-Bravo & Figueras, 2020). More than 30 different *Aeromonas* species that threaten various environmental niches and human health have been identified (Hoel et al., 2019; Fernandez-Bravo & Figueras, 2020). *Aeromonas veronii* bv.sobria, *Aeromonas hydrophila*, and *Aeromonas caviae* (*A. caviae*) are pathogenic to humans and are responsible for a broad spectrum of human intestinal and extra-intestinal infections (Janda & Abbott, 2010). Gastroenteritis associated with *Aeromonas* infection is primarily caused by the consumption of contaminated water or foods. Extra-intestinal infections in humans include biliary tract infection, urinary tract infection, soft-tissue infection, peritonitis, pneumonia, and bacteremia. Previous studies have indicated that *Aeromonas* causes infections in both immunosuppressed and immunocompetent persons (Dwivedi et al., 2008; Chuang et al., 2011). Individuals with underlying diseases, such as liver cirrhosis, malignancies, and biliary tract diseases, are at a higher risk of infection when exposed to the pathogens (Janda & Abbott, 2010; Chao et al., 2013).

*A. caviae* has been proved to reside in the human gastrointestinal tract and is considered a significant etiological agent of gastrointestinal infections. Although intestinal infections caused by *A. caviae* may be self-limited, invasive extra-intestinal infections in immunocompromised individuals or patients with severe underlying diseases may be life-threatening or even fatal. The increasing number of extra-intestinal infections worldwide poses severe threats to public health (Luo et al., 2022; Xu et al., 2022). A study in Japan found *A. caviae* to be the most frequent pathogen causing *Aeromonas* bacteremia (Kimura et al., 2013). According to Lamy et al., *A. caviae* and *A.veronii* were the most common *Aeromonas* species causing bacteremia and gastroenteritis in France (Lamy et al., 2009). Chen et al. reported that *A. caviae* predominated in primary bacteremia and biliary tract infections in southern Taiwan (Chen et al., 2021). However, epidemiological characteristics, antimicrobial resistance profiles, virulence genes and phyloevolution of *A. caviae* causing extra-intestinal infections remain unclear.

The present study aims to investigate the epidemiological characteristics of extra-intestinal infections caused by *A. caviae* and to assess the antimicrobial resistance profiles and virulence genes to provide relevant microbiological data as the basis for effective strategies in the prevention and control of drug resistance. Phyloevolution analysis was subsequently conducted to understand the evolutionary path of *A. caviae* and the relationship between species. 47 clinical *A. caviae* causing extra-intestinal infections within a 4-year period were collected at the First Medical Center of Chinese PLA General Hospital. *Aeromonas* spp. were identified, and virulence and resistance genes were detected through whole-genome sequencing (WGS). Phylogenetic characteristics of 568 strains, including 521 strains obtained from the National Center for Biotechnology Information (NCBI) database and 47 clinical strains in our study, were analyzed.

## Materials and methods

### Data collection

A retrospective study of 47 clinical *A. caviae* isolated from 46 patients with extra-intestinal infections from 2017 to 2020 at the First Medical Center of Chinese PLA General Hospital, a 3000-bed teaching hospital, was performed. Clinical data of 46 patients, including demographics, source of infection, underlying diseases, microbiological data, and respective outcomes, were retrieved from medical records. Diagnosis of bacteremia, biliary tract infection, urinary tract infection, pneumonia, and peritonitis was based on clinical, bacteriological, and radiological investigations.

### Bacterial strains and species identification

Clinical isolates were obtained from patients with *A. caviae* extra-intestinal infections. The clinical samples of sputum and urine were plated on the blood agar, and then cultured at 37°C for 24 h to obtain the single colony. Blood, ascites, and bile were inoculated into BACTEC culture bottles using the BACT/ALERT 3D system (BioMerieux, Lyon, France). The suspicious Gram-negative bacteria were characterized by positive oxidase test, D-glucose fermentation, motility test, absence of growth in 6.5% sodium chloride, resistance to the vibriostatic agent O/129 (150 ug), and then identified using matrix-assisted laser desorption/ionization-time of flight mass spectrometry (MALDI-TOF/MS) (Bruker, Bremen, Germany). Single colonies were mixed with matrix solution, dried completely, and then MALDI-TOF/MS was tested according to the manufacturer’s protocols. Results were evaluated using an identification database and exported for local preservation and statistical analysis. An appraisal credibility score of > 95% was considered reliable (Jamal et al., 2014). Final species identification was confirmed by WGS. *A. caviae* isolates were stored in 20% glycerol at -70°C for subsequent studies.

### Antimicrobial susceptibility testing

AST was performed by the broth microdilution method using the VITEK 2 Compact System (BioMerieux, Lyon, France). The antimicrobial agents tested included piperacillin/tazobactam (TZP), cefoxitin (FOX), cefuroxime (CXM), ceftazidime (CAZ), cefotaxime (CTX), cefepime (FEP), imipenem (IPM), meropenem (MPN), amikacin (AMK), gentamicin (GEN), ciprofloxacin (CIP), levofloxacin (LEV), tetracycline (TE), trimethoprim/sulfamethoxazole (STX), chloramphenicol (C), and aztreonam (ATM), The minimum inhibitory concentrations (MICs) were interpreted according to the recommendations of the Clinical and Laboratory Standards Institute (CLSI) for *Aeromonas* spp. (Institute, 2015) *E.coli* ATCC25922 was used as the quality-control strain. Multidrug resistance (MDR) was defined as non-susceptibility to at least one agent in three or more antimicrobial categories (Magiorakos et al., 2012).

### Whole-genome sequencing and analysis

Genomic DNA of *A. caviae* was extracted using the QIAamp DNA Mini Kit (Qiagen, Hilden, Germany) according to the manufacturer’s protocol. Purified DNA of all 47 A*. caviae* strains underwent WGS using the Illumina HiSeq (Illumina, San Diego, California) platform. Sequence reads were assembled using the SOAPdenovo software (version 2.04) (Li et al., 2010). Antimicrobial resistance genes were identified using the Comprehensive Antibiotic Resistance Database (CARD) (Alcock et al., 2020). We used the basic local alignment search tool (BLAST) to detect antimicrobial resistance genes with more than 70% coverage and 80% identity in each genome sequence. BLAST was used to identify virulence genes on the virulence factor database (VFDB) (https://www.mgc.ac.cn/VFs/main.htm).

### Phylogenetic analysis

Based on the global distribution of *A. caviae*, we retrieved the assembled data of 521 A*. caviae* strains from the NCBI database (44 from patients, 181 from animals, 224 from environment, and 72 strains with no detailed source informations). *A. caviae* strain AP022254.1 was used as the reference for comparison. Reads were mapped using Burrow-Wheeler Alignerg (BWA, v0.7.12), and single nucleotide polymorphisms (SNPs) were identified using SAMtools (v1.3) to obtain mutation site information. A General Feature Format (Gff) file of each genome was generated using the genome annotation program Prokka (v1.11), then used to obtain the alignment of core genes on Roary (v3.11). Chromosomal SNP alleles were concatenated for each strain to generate a multiple alignment of all SNPs, and RAxML (v8.2.4) was run using the general time-reversible (GTR) model with a gamma distribution to construct a maximum likelihood phylogenetic tree. Average nucleotide identities (ANIs) among strains found in China and those found outside of China were calculated using JSpeciesWS to evaluate genome similarity.

### Statistical analysis

All data were analyzed using SPSS 20.0 (SPSS Inc., Chicago, IL, USA). Continuous variables were presented as mean ± standard deviation (SD).

## Results

### Epidemiological data of patients infected with *A. caviae*

A total of 47 clinical isolates were obtained from 46 patients with extra-intestinal *A. caviae* infections at the First Medical Center of Chinese PLA General Hospital from 2017 to 2020. Two strains, S122 and S128, were isolated from different specimens of the same patient with a separation interval of 48 days. The patients had a mean age ± standard deviation of 68.9 ± 13.7 years and were predominantly male (male to female ratio, 3.2). More than 60% were elderly patients aged ≥65 years (63%, 29/46). Hepatobiliary diseases (57%, 26/46) and malignancies (30%, 14/46) were two common underlying diseases. The most common source of infection was biliary tract infection (41%, 19/46), followed by bacteremia (26%, 12/46), pneumonia (17%, 8/46), urinary tract infection (15%, 7/46) and peritonitis (2%, 1/46). Among the 46 patients, two patients died in hospitalization, and five who were given poor prognoses died after discharge, resulting in an in-hospital mortality rate of 4%. 21 patients were co-infected with other bacteria, including *Enterococcus* spp. (8 cases), *Escherichia coli* (8 cases), *Klebsiella* spp. (4 cases), *Pseudomonas aeruginosa* (4 cases), *Citrobacter* spp. (2 cases), *Acinetobacter* spp. (1 case), and 5 patients were infected with two or three pathogens (**Table 1** and **Table S1**).

**Table 1:** Epidemiological data of 46 patients with extra-intestinal *A. caviae* infection from 2017 to 2020.

	No (%) of patients (n=46)
Demographic characteristic	
Age, years (mean ± standard deviation)	68.9 ± 13.7
Age, ≥65 years	29 (63%)
Gender, male	35 (76%)
Underlying diseases	
Malignancies	14 (30%)
Hepatobiliary diseases	26 (57%)
Diabetes mellitus	2 (4%)
Liver cirrhosis	1 (2%)
Kidney disease	2 (4%)
Source of infection	
Bacteremia	12 (26%)
Biliary tract infection	19 (41%)
Urinary tract infection	7 (15%)
Pneumonia	8 (17%)
Peritonitis	1 (2%)
Co-infected with other bacteria	
*Enterococcus* spp.	8 (17%)
*Escherichia coli*	8 (17%)
*Klebsiella* spp.	4 (9%)
*Pseudomonas aeruginosa*	4 (9%)
*Citrobacter* spp.	2 (4%)
*Acinetobacter* spp.	1 (2%)
Outcome	
In-hospital mortality	2 (4%)
Overall mortality	7 (15%)

### Antimicrobial susceptibility profiles

Antimicrobial susceptibility testing revealed the resistance rates to antibiotics were 38% (18/47) for cefoxitin, 34% (16/47) for cefuroxime, 32% (15/47) for cefotaxime, 30% (14/47) for tetracycline, 26% (12/47) for ceftazidime, 23% (11/47) for chloramphenicol, 21% (10/47) for cefepime, and 21% (10/47) for trimethoprim-sulfamethoxazole Most strains were susceptible to aminoglycosides and carbapenems (**Table 2**). 36% (17/47) of the strains showed susceptibility to all antimicrobial drugs tested. 40% (19/47) of the isolates were identified as MDR strains, of which 11 were from bile, 5 were from urine, 2 were from sputum, and 1 was from bloodstream. Moreover, we identified two strains resistant to carbapenems (**Table S2**).

**Table 2:** Antimicrobial susceptibility patterns of 47 *A. caviae* strains iaolated from extra-intestinal infections.

Antimicrobial agent	R^a^ [n (%)]	I [n (%)]	S [n (%)]
Piperacillin/tazobactam	4 (9)	5 (11)	38 (81)
Cefoxitin	18 (38)	3 (6)	26 (55)
Cefuroxime	15 (32)	3 (6)	29 (62)
Ceftazidime	12 (26)	0 (0)	35 (74)
Cefotaxime	16 (34)	0 (0)	31 (66)
Cefepime	10 (21)	1 (2)	36 (77)
Imipenem	1 (2)	1 (2)	45 (96)
Meropenem	2 (4)	0 (0)	45 (96)
Amikacin	0 (0)	1 (2)	46 (98)
Gentamicin	2 (4)	0 (0)	45 (96)
Ciprofloxacin	8 (17)	3 (6)	36 (77)
Levofloxacin	4 (9)	3 (6)	40 (85)
Trimethoprim-sulfamethoxazole	10 (21)	0 (0)	37 (79)
Tetracycline	14 (30)	0 (0)	33 (70)
Chloramphenicol	11 (23)	1 (2)	35 (74)
Aztreonam	7 (15)	1 (2)	39 (83)

a R, Resistant; I, Intermediate; S, Sensitive.

### Antibiotic resistance genotypic and phenotypic characteristic

**Figure 1:**
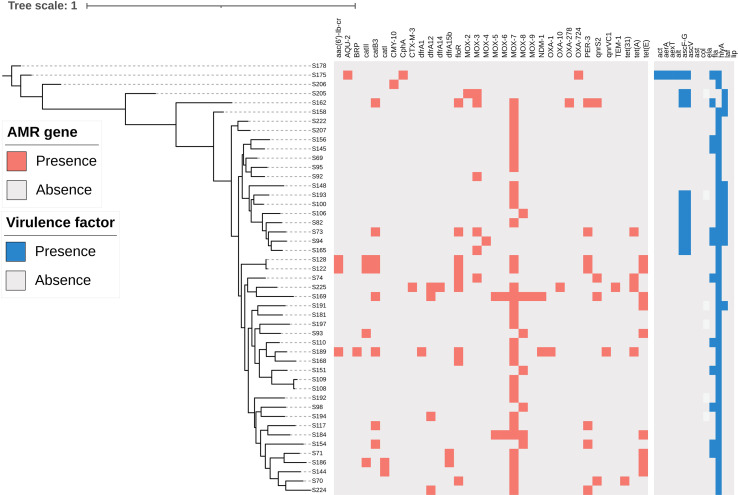
Construction of phylogenetic tree of 47 A*. caviae* strains. Antimicrobial resistance (AMR) genes were detected with more than 70% coverage and 80% identity in each genome using BLAST. Virulence genes were identified by blasting the VFDB database. Red and blue represented the antimicrobial resistance genes and virulence genes, respectively.

The antimicrobial resistance genes of *A. caviae* were shown in **Figure 1** and **Table S2**. 24 out of 47 isolates were found to be non-susceptible to cephalosporins and cephamycins, and all of which carried β-lactamase gene, including *bla*_MOX_, *bla*_PER-3_, *bla*_OXA_, *bla*_NDM_, and *bla*_CphA_. Most of the isolates (94%, 44/47) carried *bla*_MOX_, and 7 isolates carried *bla*_PER-3_. However, the AmpC β-lactamase gene, *bla*_AQU-2_, was present only in S169. The metallo-β-lactamase (MBL) gene, *bla*_CphA_ and *bla*_BRP_, was detected in S175 and S189, respectively. Another MBL gene, *bla*_NDM_, was present in S169 and S189, which exhibited carbapenem resistance. However, *bla*_CphA_ gene present in S175 was not associated with carbapenem resistance. Class A extended-spectrum β-lactamases (ESBLs) genes, *bla*_CTX-M-3_ and *bla*_TEM-1_, were simultaneous present in S225. Oxacillin-hydrolyzing (OXA)-type Class D β-lactamase encoding genes, *bla*_OXA-278_, *bla*_OXA-724_, *bla*_OXA-1_, *bla*_OXA-10_, which confer resistance to cephalosporins, were detected in S162, S175, S189, S225, respectively. The plasmid-mediated quinoloneresistance genes *qnrS2*, *qnrVC*, *aac(6’)-Ib-cr* were detected in 7 isolates, and the presence of both genes *aac(6’)-Ib-cr* and *qnrS2* was observed in S189. Most of the isolates (86%, 6/7) carrying quinolone resistance genes manifested corresponding resistance phenotypes. Tetracycline resistance genes *tet(A)*, *tet(E)*, *tet(31)* were detected in 14 isolates, all of which manifested tetracycline resistance phenotypes. All 7 strains harbored sulfonamide resistance genes *dfrA1*, *dfrA12*, *dfrA14*, *dfrA15b* manifested resistance to trimethoprim-sulfamethoxazole. 15 strains harbored the chloramphenicol resistance genes *floR*, *catB3*, *catII*, and *catI*, 80% (12/15) of which were non-susceptible to chloramphenicol.

### Distribution of virulence genes in *A.caviae* strains

We investigated representative 13 virulence genes in these clinical strains, including aerolysin (*aerA*), heat-stable cytotonic enterotoxin (*ast*), heat-labile cytotonic enterotoxin (*alt*), cytotoxic enterotoxin (*act*), hemolysin (*hlyA*), phospholipase (*lip*), type III secretion system components (*ascV* and *ascF-G*), flagellin (*fla*), elastase (*ela*), lateral flagella (*laf*), ADP-ribosyltransferase toxin (*aexT*), collagenase (*col*). The distribution of virulence genes in *A. caviae* was shown in **Figure 1** and **Table S3**. Most of the strains (98%, 46/47) carried at least one of the virulence genes. Of these strains, 94% (44/47) carried *hlyA*, followed by *fla* (26%, 12/47), *laf* (23%, 11/47), *ascV* (21%, 10/47), *ascF-G* (21%, 10/47), and three or more virulence genes were detected in 21% (10/47) of clinical isolates. The genes encoding *ast*, *lip*, *ela*, and *col* were not detected in the studied strains. Analysis of the gene profiles based on the distribution of the 13 virulence genes revealed 9 virulence patterns summarized in **Table 3**. The most predominant patterns observed were *hlyA* (53%, 25/47) *and hlyA*/*fla* (17%, 8/47). Only S175 carried several virulence genes, including *aerA*, *alt*, *act*, *hlyA*, *ascV*, *ascF-G*, *fla*, and *aexT*.

**Table 3:** Virulence gene patterns of 47 A*. caviae* strains.

Virulence gene patterns	No (%) of strains	Strain number
*hlyA*	25 (53%)	69, 70, 92, 93, 95, 108, 109, 117, 122, 128, 144, 168, 169, 181, 184, 186, 189, 192, 194, 197, 206, 207, 222, 224, 225
*hlyA*/*fla*	8 (17%)	71, 74, 98, 110, 145, 151, 154, 156
*hlyA*/*ascV*/*ascF-G*/*laf*	4 (9%)	82, 100, 106, 193
*hlyA*/*laf*	3 (6%)	148, 158, 191
*hlyA*/*ascV*/*ascF-G*/*fla*/*laf*	2 (4%)	73, 94
*ascV*/*ascF-G*/*fla*/*laf*	1 (2%)	162
*hlyA*/*ascV*/*ascF-G*	1 (2%)	165
*ascV*/*ascF-G*/*laf*	1 (2%)	205
*aerA*/*alt*/*act*/*hlyA*/*ascV*/*ascF-G*/*fla*/*aexT*	1 (2%)	175

S178 did not carry any virulence genes.

### Phylogenetic analysis

**Figure 2:**
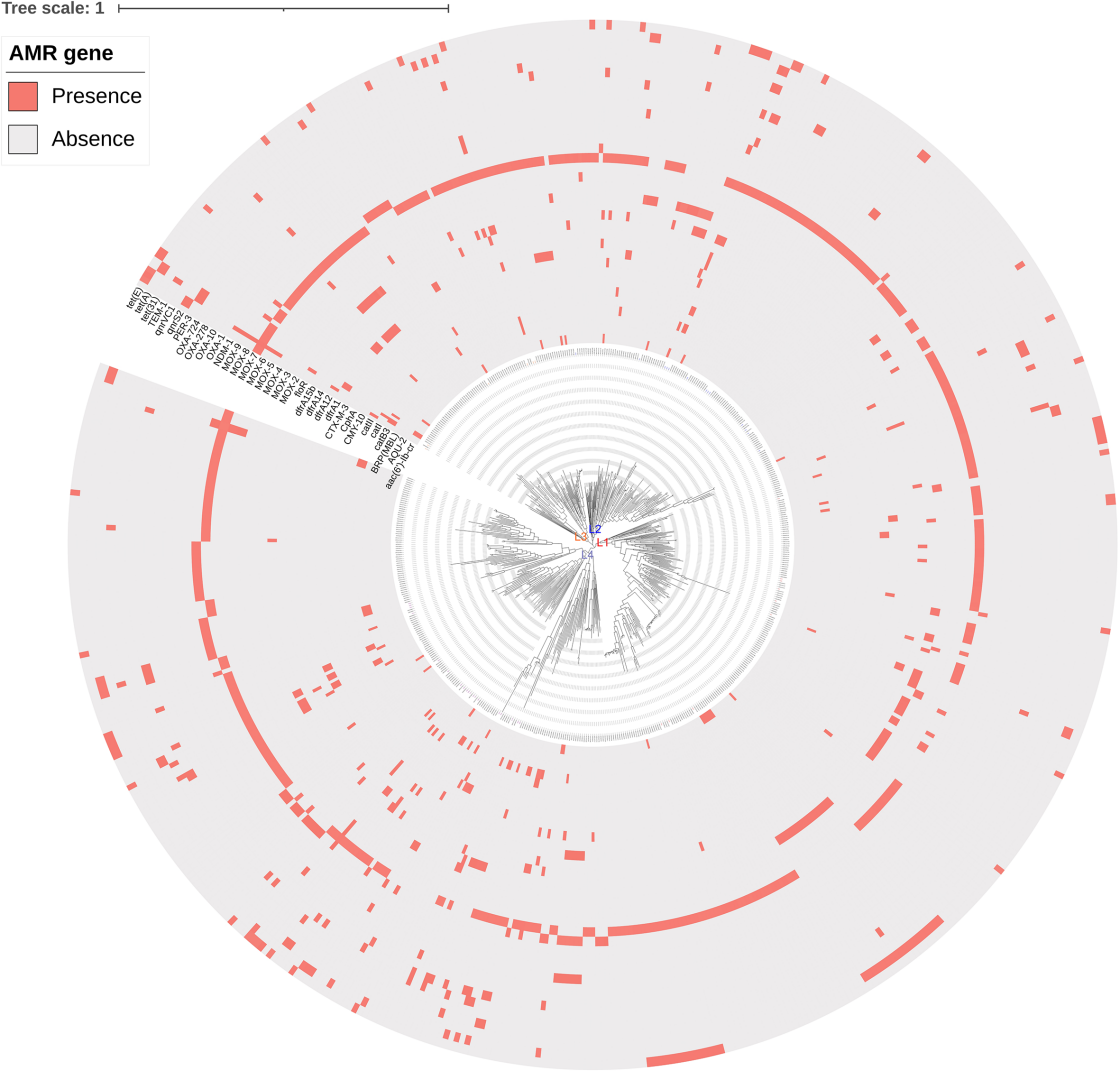
Phylogenetic tree of 568* A. caviae* strains. Maximum likelihood phylogenetic tree was constructed using RAxML (v8.2.4). The 47 isolates in L1, L2, L3, and L4 clusters were marked in red, blue, orange, and purple color, respectively. AMR gene: antimicrobial resistance gene.

Phylogenetic analysis was performed on a total of 568 strains, including 521 strains reported between 2014 and 2021 from NCBI database (**Table S4**) and 47 clinical strains in our study, and a phylogenetic tree with 243,480 SNP loci was generated (**Figure 2**). All 568 strains were divided into four major clusters, of which L1 cluster had a low genetic relationship, L2 cluster contained less drug resistance genes, L3 and L4 cluster contained more drug resistance genes. Phylogenetic analysis showed that 47 clinical strains were distributed among the four clusters (**Figure 2** and **Table S5**). The ANI between S108 and S109, which were isolated with a separation interval of 14 days from different departments on the same floor, was 100%.

## Discussion

In this study, it was found that more than 60% of extra-intestinal infections caused by *A. caviae* developed in elderly patients aged ≥ 65 years, most having immunocompromised conditions or underlying diseases, including hepatobiliary diseases, malignancies, diabetes mellitus, and renal disease. Furthermore, over 50% of patients with *A. caviae* infection had coexisting hepatobiliary diseases, including biliary stones, obstructive biliary disease, gallbladder or biliary tract tumor, and liver cancer, of which 8 patients suffered from bloodstream infections, 16 patients had biliary tract infections, and 1 patient had biliary tract infection followed by bloodstream infection. The high occurrence of infection with *A. caviae* may be attributable to biliary tract obstruction or stasis with increased intraductal pressure during hepatobiliary diseases. Moreover, translocation of *A. caviae* present in the gastrointestinal tract into intrahepatic and extrahepatic bile ducts, as well as hepatic veins and lymphatics, remain a possible cause of biliary tract infection and bacteremia (An et al., 2021). Polymicrobial infections occurred in 14 of 19 patients with *A. caviae* biliary tract infection, demonstrating that patients with biliary tract infection have a higher risk of developing polymicrobial infections. Malignant tumor patients who have received chemotherapy are more vulnerable to foodborne infections due to the disruption of their intestinal mucosal barrier, and the ensuing neutropenia makes the host susceptible to opportunistic infections (Baden et al., 2016). Consistent with previous studies, malignancy was found to be a common underlying disease in our study (Wu et al., 2015; Chen et al., 2021). Furthermore, liver cirrhosis has been recognized as a predisposing condition associated with *Aeromonas* bacteremia in regions with a high prevalence of chronic hepatitis (Tang et al., 2014). However, none of the 12 patients with *A. caviae* bacteremia had liver cirrhosis, and only one patient with liver cirrhosis developed urinary tract infection. Although *A. caviae* has been considered the primary culprit of bacteremia and biliary tract infections, *A. caviae* has also been reported in a few cases of urinary tract infections (Chao et al., 2012). In our study, more than 15% (7/46) of patients suffered from urinary tract infection, 5 of which are immunocompromised or undergoing invasive therapeutical procedures. Therefore, *A. caviae* should be considered a possible pathogen in immunocompromised patients, individuals suffering from underlying diseases, elderly patients, and patients undergoing invasive medical procedures.

CLSI has recommended third and fourth generation cephalosporins, fluroquinolones, and trimethoprim-sulfamethoxazole for the treatment of infections with *Aeromonas* spp. To date, there are only a few studies on the antimicrobial resistance of extra-intestinal *A. caviae* isolates, with one study reporting 15%-30% resistance rates for ceftazidime, cefepime, and ceftriaxone from 2012 to 2017 (Yang et al., 2019). Results of our study demonstrated that the resistance rate for cefotaxime was over 30%, and the resistance rates for ceftazidime, cefepime and trimethoprim-sulfamethoxazole were over 20%. Although the incidence of MDR reached to 40%, resistance rates to several antibiotics remained below 10%, including piperacillin-tazobactam, levofloxacin, gentamicin, amikacin, imipenem, and meropenem. These findings provide guidance for selecting appropriate empirical treatment before obtaining AST results.

In the present study, *bla*_MOX_ was detected in more than 90% of isolates. This result concurs with previous findings that AmpC β-lactamases genes are species-specific to *Aeromonas* spp. and that all *A. caviae* isolates carry *bla*_MOX_ (Fosse et al., 2003; Wu et al., 2015). However, only 50% (22/44) of the strains carrying *bla*_MOX_ exhibited the cephalosporins or cephamycins non-susceptibility. Thus, the presence of the *bla*_MOX_ gene was not associated with corresponding antibiotics non-susceptibility as reported previously (Walsh et al., 1997).

The class A extended-spectrum β-lactamases (ESBLs) gene *bla*_PER-3_ shared 99% identity with *bla*_PER-1_ and was initially identified within an isolate of *Aeromonas punctata* in France adjacent to a copy of ISCR1 (insertion sequence common regions, ISCRs), and then detected in two *A.caviae* isolates from a medical center in Taiwan and *A.veronii* isolated from chicken cloaca (Toleman et al., 2006; Wu et al., 2011; Wang et al., 2020). We found 7 *bla*_PER-3_-producing *A. caviae* isolates, all of which were MDR strains. Previous research revealed that the horizontal transfer of genetic elements, such as plasmids and integrons, could lead to an increased incidence of MDR among environmental *Aeromonas* isolates (Jacobs & Chenia, 2007). The spread of *Enterobacteriaceae* carrying the *bla*_PER-1_ gene as a chromosomal insert has been reported in Europe (Perilli et al., 2007). Therefore, caution should be exercised to prevent transmission of drug resistance genes between *Aeromonas* spp. by genetic elements.

Carbapenem resistance has been detected in *A.hydrophila* and *A.veronii* isolates, but is rarely found in *A. caviae* strains (Chen et al., 2012). However, recent studies have found that plasmid-encoded or non-plasmid encoded *bla*_NDM-1_, *bla*_OXA-181_, *bla*_VIM_, and *bla*_KPC-2_ genes contribute to carbapenem resistance in clinical *A. caviae* isolates, indicating the possibility of evolution and transmission of resistance genes in clinical strains (Adler et al., 2014; Anandan et al., 2017; Tang et al., 2020; Luo et al., 2022; Xu et al., 2022). In this study, two strains isolated from urine harbored *bla*_NDM-1_ and exhibited carbapenem resistance, one of which co-harbored of *bla*_CTX-M-3_ and class D β-lactamases gene *bla*_OXA-1_, which conferred resistance to third or fourth generation cephalosporins. The emergence of MDR strains poses a threat that challenges the diagnosis, clinical treatment, and control of infectious diseases. Studies on the genetic characteristics of these two strains are being conducted to elucidate the role of mobile genetic elements, such as plasmids and integrons, in the transmission of resistance.

According to one study, the AmpC β-lactamase gene *bla*_AQU-2_ was found only in *A. hydrophila* and *A. jandaei* strains isolated from chicken rinse (Wang et al., 2021). The MBL gene *bla*_CphA_, which is involved in the intrinsic resistance of *Aeromonas* to carbapenems, was found in the majority of *A.hydrophila* isolates but rarely detected in *A. caviae* strains (Wu et al., 2015; Wu et al., 2019). In our study, one *bla*_CphA_-carrying *A. caviae*, which co-harbored both *bla*_AQU-2_ and the class D β-lactamases gene *bla*_OXA-724_, conferred resistance to third generation cephalosporins, but did not exhibit carbapenem resistance *in vitro*, possibly attributed to the difficulty in detecting carbapenemase activity of carbapenemase hydrolyzing Aeromonas (CphA) through conventional AST, or genetic modifications that alter the expression of CphA (Wu et al., 2012).

To date, the virulence and pathogenic mechanism of *Aeromonas* remain obscure. Many virulence factors, including cytotoxins, enterotoxins, hemolysins, cell surface structures, lipases, proteases, aerolysins, and secretory systems, contributed to survival, environmental adaptation, and disease pathogenesis (Li et al., 2015; Wu et al., 2019; Zhou et al., 2019; Chen et al., 2021; Sun et al., 2021). Previous studies observed lower virulence and lower fatality rates in *A. caviae* compared to other *Aeromonas* spp. (Wu et al., 2015; Wu et al., 2019). However, findings from several studies on virulence genes and virulence phenotype of *A. caviae* remain controversial. In the present study, we revealed 9 virulence patterns composed of one to eight genes. Additionally, we found that the majority of the *A. caviae* strains carried at least one of the virulence genes, and three or more virulence genes were detected in more than 20% of these isolates. Pablos et al. reported that *A. caviae* infrequently carried *aerA*, *hlyA*, and *ast* genes (Pablos et al., 2010). Wu et al. found that the major genotype in clinical *A. caviae* isolates was *lip*, *col*, and *ela* (Wu et al., 2019). However, our finding contradicts the results of previous studies. More than 90% of isolates carried *hlyA*, and none carried *lip*, *col*, or *ela*. In accordance with previous research, our study showed that *ast* was absent in clinical *A. caviae* isolates (Aguilera-Arreola et al., 2007). Research has demonstrated that flagella glycosylation in *A.hydrophila* plays a vital role in Hep-2 cell adhesion and biofilm formation (Merino et al., 2014). Previous study showed that *fla* and *laf* were highly prevalent in *A. caviae* isolated from human faeces (Santos et al., 2011). In a recent study, 92.3% of *A. caviae* isolated from clinical specimens harbored *fla* (Miyagi et al., 2021). This study deteced *fla* and *laf* in only 26% and 23% of extra-intestinal *A. caviae* isolates, respectively. The type III secretion system (TTSS) of *Aeromonas* has been proved to play an essential role in pathogenicity (Yu et al., 2004). Studies found *ascV* and *ascF-G* in only a few extra-intestinal *A. caviae* isolates (14.3%, 2/14) (Chacon et al., 2004). Chen et al. demonstrated that infection with *ascF-G Aeromonas* was associated with mortality (Chen et al., 2021). We found that more than 20% of extra-intestinal *A. caviae* isolates (21%, 10/47) possesed these TTSS encoding genes, but only one patient, who was infected with *A. caviae* harboring *ascV* and *ascF-G*, died in the hospital.

One patient (case 46) infected with *A. caviae* carrying 8 virulence genes was cured after 14 days of hospitalization. However, another patient (case 18) infected with *A. caviae* possessing only hemolysin encoding gene *hlyA* died 45 days after admission. The significant difference between the two patients was that the latter had liver cancer. Our work showed that the overall mortality of extra-intestinal *A. caviae* infection was 15% (7/46). Additionally, we found that most of the strains (86%, 6/7) isolated from the 7 patients who died harbored only one virulence coding gene *hlyA*, and 5 of the 7 patients suffered from cancer. It is possible that host factors, such as underlying diseases, prolonged hospitalization, and not the virulence of *A. caviae* itself, are the main causes of death. We did not find an association between virulence genes and the pathogenicity of extra-intestinal *A. caviae* stains, suggesting the need for further research to identify specific virulence gene and the mechanism of pathogen-host interaction in extra-intestinal *A. caviae* infection.

In this retrospective study, we analyzed the genomic evolutionary characteristics of *A. caviae* based on 47 clinical isolates and 521 public strains available in the NCBI database. The genetic relationships of the 47 strains were identified, and cluster analysis indicated differences in SNPs among the 47 strains. These isolates had low genetic relationships, which might be due to the long separation interval of these strains. Genomic evolutionary analysis showed that stains in L3 and L4 contained more drug resistance genes than those in L2, presumably because changes in some key SNPs determined the subsequent evolution, and some drug resistance genes were inserted during the evolution. A high degree of homology between S108 and S109, but low homology between other stains indicated that there existed a risk of nosocomial transmission, but no clustering of hospital-onset infections was noted.

## Conclusion

The epidemiological characteristics, antimicrobial resistance profiles, virulence genes, and phylogenetic traits described in this study strengthen our understanding of *A.caviae* strains that cause extra-intestinal infections. It may contribute to the management of extra-intestinal infections as well as the prevention and control of drug resistance.

## Data availability statement

The data presented in the study are deposited in the National Library of Medicine repository (https://www.ncbi.nlm.nih.gov/sra/PRJNA902936), accession number PRJNA902936.

## Ethics statement

This study involving human participants was reviewed and approved by the Ethics Committee of First Medical Center of Chinese PLA General Hospital.

## Author contributions

YS, L-FW, D-XS, and YC designed the study. KZ, SL, LG, L-YY, and JG performed the experiments and interpreted the data. YS, YC, and L-FW wrote the first draft of the paper. YS, YC, L-FW and D-XS reviewed and approved the final report. All authors contributed to the article and approved the submitted version.
