# Supplementary material for: Epidemiological characteristics, virulence potential, antimicrobial resistance profiles, and phylogenetic analysis of Aeromonas caviae isolated from extra-intestinal infections
Source: Front Cell Infect Microbiol. 2023 Feb 24;13:1084352. doi: 10.3389/fcimb.2023.1084352 (PMC9999030; doi:10.3389/fcimb.2023.1084352)
Supplement: Supplementary file 1 [file Table_1.docx]

**Table S1 Demographics of 46 patients with extra-intestinal *A. caviae* infections**

| **Case number** | **Strain number** | **Age/gender** | **Specimen type** | **Time of isolation** | **Underlying disease** | **Co-infection** | **Outcome** |
| --- | --- | --- | --- | --- | --- | --- | --- |
| 1 | S69 | 87/M | Blood | 2017/7/6 | Cholangitis, pancreatic cancer, biliary tract obstruction | No | Died after discharge |
| 2 | S70 | 60/F | Urine | 2017/7/6 | Connective tissue disease, cirrhosis, autoimmune hemolytic anemia | No | Survival |
| 3 | S71 | 92/F | Sputum | 2017/7/10 | Hypertension, coronary atherosclerotic heart disease | No | Survival |
| 4 | S73 | 64/M | Urine | 2017/7/20 | Postoperative of ureteral stones | *Escherichia coli* | Survival |
| 5 | S74 | 72/F | Bile | 2017/7/20 | Cholangitis, bile duct stones | *Escherichia coli, Pseudomonas aeruginosa* | Survival |
| 6 | S82 | 50/M | Urine | 2017/9/6 | Urethral atresia, post-cystostomy | No | Survival |
| 7 | S92 | 62/F | Bile | 2017/11/9 | Cholangitis, bile duct stones | No | Survival |
| 8 | S93 | 66/M | Blood | 2017/11/14 | Bile duct stones | *Escherichia coli, Klebsiella* spp, *Enterococcus* spp | Survival |
| 9 | S94 | 41/M | Bile | 2017/12/6 | Choledochal cyst complicated by bile duct stones | *Enterococcus* spp | Survival |
| 10 | S95 | 66/M | Ascites | 2017/12/7 | Chronic renal failure, peritonitis, diabetes, hypertension, renal anemia | No | Survival |
| 11 | S98 | 69/F | Bile | 2017/12/16 | Cystic dilatation of bile duct | *Enterococcus* spp, *Klebsiella* spp | Survival |
| 12 | S100 | 53/F | Blood | 2018/1/2 | Lung cancer | No | Survival |
| 13 | S106 | 83/M | Blood | 2018/4/25 | Lung cancer, hypertension, diabetes | No | Died in hospital |
| 14 | S108 | 63/F | Bile | 2018/5/26 | Bile duct stones, cholangitis | No | Survival |
| 15 | S109 | 48/F | Bile | 2018/6/10 | Cholangitis, bile duct stones | *Enterococcus* spp | Died after discharge |
| 16 | S110 | 43/F | Bile | 2018/6/9 | Choledochal cyst, biliary anastomotic obstruction | *Escherichia coli* | Survival |
| 17 | S117 | 62/M | Bile | 2018/7/16 | Cholangiocarcinoma, obstructive jaundice | No | Survival |
| 18 | S122 | 56/M | Bile | 2018/8/9 | Liver cancer, obstructive jaundice | No | Died in hospital |
|  | S128 |  | Blood | 2018/9/26 |  |  |  |
| 19 | S144 | 75/M | Sputum | 2018/11/10 | Esophageal cancer | *Pseudomonas aeruginosa* | Died after discharge |
| 20 | S145 | 90/F | Sputum | 2018/11/11 | Gallbladder stones, cholecystitis, pancreatitis | No | Survival |
| 21 | S148 | 62/M | Bile | 2018/11/24 | Biliary mucinous cystadenoma | *Escherichia coli, Enterococcus* spp | Survival |
| 22 | S151 | 87/M | Blood | 2018/12/24 | COPD | No | Survival |
| 23 | S154 | 68/M | Sputum | 2019/2/12 | Gastrointestinal hemorrhage, hypertension, renal insufficiency | No | Survival |
| 24 | S156 | 75/M | Urine | 2019/3/30 | Vertebrobasilar artery stenosis | No | Survival |
| 25 | S158 | 87/M | Blood | 2019/4/21 | Gallbladder stones, coronary atherosclerotic heart disease | No | Survival |
| 26 | S162 | 85/M | Urine | 2019/5/23 | Bladder cancer and postoperation | *Enterococcus* spp | Survival |
| 27 | S165 | 89/M | Blood | 2019/6/2 | Gallbladder stones | No | Survival |
| 28 | S168 | 75/F | Bile | 2019/6/17 | Gallbladder stones | *Enterococcus* spp | Survival |
| 29 | S169 | 73/M | Urine | 2019/6/28 | Post-prostate paracentesis, urinary tract infection | No | Survival |
| 30 | S175 | 43/M | Bile | 2019/8/4 | Choledochal cyst complicated by bile duct stones | *Klebsiella* spp | Survival |
| 31 | S178 | 87/M | Sputum | 2019/8/24 | Pulmonary interstitial fibrosis, pulmonary nodules | No | Survival |
| 32 | S181 | 72/M | Bile | 2019/9/15 | Pancreatic head carcinoma | *Klebsiella* spp, *Citrobacter* spp | Survival |
| 33 | S184 | 63/M | Bile | 2019/9/21 | Rectal cancer | No | Survival |
| 34 | S186 | 65/M | Sputum | 2019/10/21 | Cardiac insufficiency | No | Died after discharge |
| 35 | S189 | 86/M | Urine | 2019/11/9 | Urinary retention, prostatic hyperplasia | No | Survival |
| 36 | S191 | 57/M | Bile | 2019/12/13 | Cholangiocarcinoma, obstructive jaundice | *Citrobacter* spp | Survival |
| 37 | S192 | 86/M | Sputum | 2019/12/18 | Coronary atherosclerotic heart disease, hypertension | *Pseudomonas aeruginosa* | Survival |
| 38 | S193 | 69/M | Blood | 2019/12/17 | Bile duct stones | No | Survival |
| 39 | S194 | 49/M | Sputum | 2019/12/27 | Left lung shadow | No | Survival |
| 40 | S197 | 82/M | Blood | 2020/3/24 | Cholangitis, bile duct stones | *Escherichia coli* | Survival |
| 41 | S205 | 72/M | Bile | 2020/7/21 | Cholangiocarcinoma, intrahepatic duct stone | *Enterococcus* spp | Survival |
| 42 | S206 | 76/M | Bile | 2020/7/23 | Cholangitis, bile duct stone | *Pseudomonas aeruginosa* | Survival |
| 43 | S207 | 70/M | Blood | 2020/8/2 | Cholangiocarcinoma, cholangio-intestinal anastomotic stricture, cholangitis | *Escherichia coli* | Survival |
| 44 | S222 | 72/M | Blood | 2020/10/26 | Cholangiocarcinoma, abdominal infection | No | Died after discharge |
| 45 | S224 | 65/M | Bile | 2020/11/4 | Cholangiocarcinoma | *Acinetobacter* spp | Survival |
| 46 | S225 | 53/M | Bile | 2020/12/2 | Cholangio-intestinal anastomotic stricture complicated by stones | *Escherichia coli* | Survival |

COPD: chronic obstructive pulmonary disease
