# Supplementary material for: Epidemiological characteristics, virulence potential, antimicrobial resistance profiles, and phylogenetic analysis of Aeromonas caviae isolated from extra-intestinal infections
Source: Front Cell Infect Microbiol. 2023 Feb 24;13:1084352. doi: 10.3389/fcimb.2023.1084352 (PMC9999030; doi:10.3389/fcimb.2023.1084352)
Supplement: Supplementary file 4 [file Table_4.docx]

**Table S4 The details of the 521 *A.caviae* strains downloaded from NCBI database**

| **Project** | **Number of strains** | **Country** | **Period of study** | **Identification method** | **Species** |
| --- | --- | --- | --- | --- | --- |
| PRJNA512885 | 3 | Brazil | 2020/12/19 | WGS | *A. caviae* |
| PRJNA685948 | 4 | Spain | 2021/2/8 | WGS | *A. caviae* |
| PRJNA664939 | 6 | USA | 2020/10/30 | WGS | *A. caviae* |
| PRJEB36872 | 2 | France | 2020/9/29 | WGS | *A. caviae* |
| PRJEB15489 | 203 | - | 2020/9/10 | WGS | *A. caviae* |
| PRJDB10296 | 1 | Japan | 2020/7/30 | WGS | *A. caviae* |
| PRJDB6962 | 44 | Japan | 2020/7/21 | WGS | *A. caviae* |
| PRJNA628854 | 2 | Poland | 2020/6/9 | WGS | *A. caviae* |
| PRJNA615899 | 1 | Brazil | 2020/4/2 | WGS | *A. caviae* |
| PRJNA607550 | 231 | Asia | 2021/4/1 | WGS | *A. caviae* |
| PRJNA566093 | 1 | Africa | 2019/10/12 | WGS | *A. caviae* |
| PRJEB31025 | 2 | United Kingdom | 2019/2/13 | WGS | *A. caviae* |
| PRJNA489399 | 1 | Canada | 2020/10/9 | WGS | *A. caviae* |
| PRJEB26597 | 2 | Tanzania | 2018/5/6 | WGS | *A. caviae* |
| PRJNA433857 | 1 | Afghanistan | 2018/7/14 | WGS | *A. caviae* |
| PRJNA292995 | 2 | Mexico | 2015/8/18 | WGS | *A. caviae* |
| PRJNA723354 | 15 | USA | 2021/4/20 | WGS | *A. caviae* |

WGS: whole genome sequencing
